# Green Tea with Rhubarb Root Reduces Plasma Lipids While Preserving Gut Microbial Stability in a Healthy Human Cohort

**DOI:** 10.3390/metabo15020139

**Published:** 2025-02-19

**Authors:** Amanda J. Lloyd, MJ Pilar Martinez-Martin, Alina Warren-Walker, Matthew D. Hitchings, Odin M. Moron-Garcia, Alison Watson, Bernardo Villarreal-Ramos, Laura Lyons, Thomas Wilson, Gordon Allison, Manfred Beckmann

**Affiliations:** 1 Department of Life Sciences, Aberystwyth University, Aberystwyth SY23 3DA, Wales, UK; mam167@aber.ac.uk (M.P.M.-M.); arw21@aber.ac.uk (A.W.-W.); aan@aber.ac.uk (A.W.); bev10@aber.ac.uk (B.V.-R.); lal8@aber.ac.uk (L.L.); tpw2@aber.ac.uk (T.W.); meb@aber.ac.uk (M.B.); 2 Faculty of Medicine Health & Life Science, Swansea University, Swansea SA2 8QA, Wales, UK; m.hitchings@swansea.ac.uk; 3 Institute of Biology, Environmental and Rural Sciences, Aberystwyth University, Aberystwyth SY23 3EB, Wales, UK; goa@aber.ac.uk

**Keywords:** *Camellia sinensis*, rhubarb root, plasma lipids, 16S rRNA amplicon sequencing, cholesterol, low-density lipoprotein, intervention trial

## Abstract

**Background/Objectives**: Cardiovascular diseases remain a leading cause of mortality and morbidity, and dyslipidaemia is one of the major risk factors. The widespread use of herbs and medicinal plants in traditional medicine has garnered increasing recognition as a valuable resource for increasing wellness and reducing the onset of disease. Several epidemiologic and clinical studies have shown that altering blood lipid profiles and maintaining gut homeostasis may protect against cardiovascular diseases. **Methods**: A randomised, active-controlled parallel human clinical trial (n = 52) with three herbal tea infusions (green (*Camellia sinensis*) tea with rhubarb root, green tea with senna, and active control green tea) daily for 21 days in a free-living healthy adult cohort was conducted to assess the potential for health benefits in terms of plasma lipids and gut health. Paired plasma samples were analysed using Afinion lipid panels (total cholesterol, LDL (low-density lipoprotein) cholesterol, HDL (high-density lipoprotein) cholesterol, triglycerides, and non-HDL cholesterol) and paired stool samples were analysed using 16S rRNA amplicon sequencing to determine bacterial diversity within the gut microbiome. **Results**: Among participants providing fasting blood samples before and after the intervention (n = 47), consumption of herbal rhubarb root tea and green tea significantly lowered total cholesterol, LDL-cholesterol, and non-HDL cholesterol (*p* < 0.05) in plasma after 21 days of daily consumption when compared with concentrations before the intervention. No significant change was observed in the senna tea group. In participants providing stool samples (n = 48), no significant differences in overall microbial composition were observed between pre- and post-intervention, even at the genus level. While no significant changes in overall microbial composition were observed, specific bacterial genera, such as Dorea spp., showed correlations with LDL cholesterol concentrations, suggesting potential microbiota-mediated effects of tea consumption. Diet and BMI was maintained in each of the three groups before and after the trial. **Conclusions**: It was found that drinking a cup of rhubarb root herbal or green tea infusion for 21 days produced beneficial effects on lipid profiles and maintained gut eubiosis without observable adverse effects in a healthy human cohort. More studies are needed to fully understand the effects of rhubarb root and green tea in fatty acid metabolism and gut microbial composition.

## 1. Introduction

The widespread use of herbs and medicinal plants in traditional medicine has gained increasing recognition as a valuable resource for increasing wellness and reducing the onset of disease. *Camellia sinensis* (tea) one of the most popular beverages worldwide, is desired not only for its taste and aroma but also for its wellness promoting properties, cultural significance, and diverse social appeal [1]. Many studies have demonstrated that tea, as well as herbal blends containing tea, shows various health functions, including antioxidant, anti-inflammatory, immuno-regulatory, anticancer, cardiovascular-protective, anti-diabetic, anti-obesity, and hepato-protective effects [2].

Cardiovascular diseases (CVDs) are diseases involving the circulatory system (heart, arteries, and veins), and includes heart conditions, hypertension, otherwise known as persistently high blood pressure, and narrowing of arteries as a result of deposition of fat, cholesterol, and other substances along the arterial walls, thus limiting the supply of blood to various part of the body and organs [3]. CVDs remain a leading cause of mortality and morbidity in the Western world. The ongoing search for factors that can reduce the prevalence of these conditions remains a prominent focus in research. Several epidemiologic and clinical studies [4,5] have consistently demonstrated that abnormal lipid profiles, characterised by high concentrations of total cholesterol, triacylglycerols, and low-density lipoprotein (LDL) cholesterol, alongside low concentrations of high-density lipoprotein (HDL) cholesterol, are significant risk factors for the development of CVDs. Elevated total cholesterol and LDL cholesterol contribute to the formation of atherosclerotic plaques, which narrow arteries and impede blood flow, ultimately increasing the risk of myocardial infarction, stroke, and other cardiovascular events [6]. Beyond the cardiovascular system, high cholesterol concentrations have been linked to other health issues, such as non-alcoholic fatty liver disease (NAFLD), which can progress to liver inflammation, fibrosis, and cirrhosis, as well as an increased risk of gallstone formation [7]. Additionally, dyslipidaemia has been associated with cognitive decline and an elevated risk of neurodegenerative diseases such as Alzheimer’s disease, potentially due to the impact of cholesterol on neuronal membrane integrity and amyloid-beta plaque formation [8]. High triglyceride concentrations further exacerbate this risk by promoting a pro-inflammatory and pro-thrombotic state within the vascular system [9]. Conversely, low HDL cholesterol concentrations are detrimental as HDL plays a protective role in cardiovascular health by facilitating reverse cholesterol transport, where cholesterol is removed from arterial walls and transported to the liver for excretion [9]. These lipid imbalances, when coupled with other factors such as hypertension, obesity, diabetes, and smoking, significantly heighten the likelihood of adverse health outcomes, underscoring the critical need for interventions aimed at improving lipid profiles to mitigate both cardiovascular and systemic health risks.

The gut microbiota plays a crucial role in maintaining host metabolic homeostasis, with its composition and functionality intricately linked to lipid metabolism and cardiovascular health [10]. Dysbiosis, or an imbalance in the gut microbiota, has been associated with dyslipidaemia and the progression of CVD through multiple mechanisms [11]. First, the gut microbiota influences bile acid metabolism, which regulates cholesterol absorption and excretion. Certain bacterial species, such as *Lactobacillus* and *Bifidobacterium*, enhance bile salt deconjugation and cholesterol assimilation, thereby lowering serum cholesterol concentrations [12]. Second, microbial metabolites such as short-chain fatty acids (SCFAs) produced by fibre fermentation can improve lipid metabolism by modulating lipid synthesis and energy homeostasis in the liver [13,14]. Moreover, specific microbial taxa are linked to lipid profiles. For instance, higher abundance of *Dorea* spp. and *Bacteroides* has been associated with favourable lipid concentrations, while an over-representation of *Firmicutes* and *Proteobacteria* is often observed in individuals with elevated cholesterol and triglycerides [10]. Additionally, pro-inflammatory metabolites such as trimethylamine-N-oxide, produced by microbial metabolism of dietary choline and carnitine, contribute to endothelial dysfunction and atherogenesis, heightening cardiovascular risk [15]. Interventions such as dietary modifications, probiotics, and prebiotics have demonstrated efficacy in reshaping the gut microbiota, improving lipid profiles, and potentially reducing cardiovascular risk [16].

Nutrition plays a vital role in shaping the intestinal microbiota, which is crucial for maintaining digestive and overall health. Dietary components such as fibre and prebiotics promote the growth of beneficial bacteria like *Bifidobacterium* and *Lactobacillus*, leading to the production of SCFAs that support gut integrity and reduce inflammation [17,18,19]. Conversely, diets high in saturated fats, refined sugars, and low in fibre can lead to dysbiosis—an imbalance in microbial communities—associated with increased intestinal permeability, systemic inflammation, and metabolic disorders [20]. Bioactive compounds, such as polyphenols in tea, fruits, and vegetables, also modulate gut microbiota by promoting beneficial species and suppressing harmful ones [21].

Data suggest that drinking green tea, rich in polyphenols, has beneficial effects, which protects against CVD by modulating gut microbiota, restoring eubiosis, and improving blood lipid profiles [4,22,23], amongst other things. Several studies have investigated the modulation effect of green tea on the gut microbiota in humans [24,25]. These findings appear to support the hypothesis that tea ingestion could favourably regulate the profile of the gut microbiome and help to offset dysbiosis triggered by obesity or high-fat diets.

Tea infusions are often enriched with herbs and botanicals, usually selected and blended for a specific health effect and taste profile. Preliminary data from Cassia species such as Senna (*Cassia acutifolia* and *Cassia angustifolia*) indicate the potential to improve blood lipid profiles [26] in rodent models and in vitro, with some evidence suggesting potential antioxidant and anti-inflammatory properties [27,28]. However, Cassia species are also known for their laxative effects [29], as they contain anthraquinones such as sennosides, which are likely responsible for the purgative effect [30]. Cassia seeds, a less-studied component, have been reported to exhibit hepatoprotective and hypolipidemic effects, potentially aiding in the prevention of metabolic disorders [31]. Rhubarb also contains anthraquinones such as sennosides and possesses a milder laxative effect [32,33]. Beyond its laxative properties, rhubarb demonstrates antioxidant, anti-inflammatory, and cholesterol-lowering effects [34,35]. It has been used traditionally to treat gastrointestinal disorders, support liver health, and manage blood glucose concentrations [35]. Interestingly, rhubarb roots, often considered waste products of rhubarb stem cultivation, contain similar amounts of active ingredients as the stalk, making them a sustainable resource for functional food development. Other botanicals such as lotus leaf (*Nelumbo nucifera*), fiveleaf gynostemma (*Gynostemma pentaphyllum*), honeysuckle flower (*Lonicera japonica*), and hawthorn fruit (*Crataegus pinnatifida*), are often added to herbal teas and have documented health benefits. Lotus leaf is known for its anti-obesity, anti-inflammatory, and lipid-lowering properties [36,37], and it may support blood circulation and lipid metabolism. Fiveleaf gynostemma, has adaptogenic effects, enhances antioxidant activity, and supports cardiovascular health [38]. Honeysuckle flower exhibits antimicrobial and anti-inflammatory activities and may support immune health [39], while also contributing to bowel health by restoration of the gut’s natural fluid balance. Hawthorn fruit has been studied for its cardioprotective effects, including blood pressure regulation, lipid-lowering properties, and antioxidant activity [39]. These botanicals that are popular as additives to tea and herbal tea drinks, alongside others such as thyme, oregano, chamomile, and mint, may contribute to healthier blood lipid profiles and improved digestive function, impacting faecal consistency and composition positively.

Although several trials have examined the effect of green tea on lipid profiles and gut microbiota in human cohorts, data on green tea with senna or rhubarb root remain limited. This study aims to investigate the combined effects of green tea with rhubarb root or senna on lipid metabolism and gut microbiota in a healthy human cohort. By assessing changes in blood lipid profiles and gut microbial composition, this research seeks to provide novel insights into the lipid-lowering potential and gut microbiome-preserving effects of these herbal tea blends. This double-blind, randomised, controlled clinical trial represents an essential step toward validating the functional benefits of herbal tea infusions in cardiovascular health and metabolic regulation.

## 2. Materials and Methods

### 2.1. Subjects

This was a double-blind, randomised, active controlled parallel human clinical trial (registered in the ISRCTN registry (https://www.isrctn.com/ accessed on 17 February 2025) under registry number ISRCTN 32761538, approved by the Research Ethics Panel at Aberystwyth University. Subjects were recruited by the Well-being and Health Assessment Research Unit (WARU) at Aberystwyth University and the study was carried out in accordance with the Declaration of Helsinki, where participants gave written informed consent. The pre-screening for the first volunteers started in May 2022 and the study was completed in May 2023. The trial was conducted at Canolfan Plas Y Sarn, Trimsaran, and the participants followed the intervention trial at home and came to the centre before and after the trial for measurements, blood collections, and questionaries.

Participants were recruited according to eligibility criteria. Inclusion criteria included consenting adults > 18 years of age; commit to fasting capillary blood collection; commit to stool sampling collection; able to refrain from taking any over-the-counter medication or herbal supplements during the diet monitoring and experimental periods; able to prepare and consume the tea during the experimental days, after the last meal/snack of the day and not to consume anything afterward; fill in diet questionnaires and stool ranking; able to inform the researcher if any antibiotics or heavy alcohol is consumed over the intervention period. Exclusion criteria included serious health conditions that require daily long-term medications (including immunosuppressants); a history or current diabetes, lung issues, gut inflammation (Crohn’s, IBD), digestive disorders; diagnosed with a serious health condition within the last 12 months; pregnant or lactating; play sports at a high level (more than 7 h/week or 1 h/day); smoker; consume high dose of alcohol > 21 unit per week for men and >14 units per week for women; food allergy/food intolerance/eating disorder or are on a specially prescribed diet. If eligible, participants were allocated to three groups using computer-generated random number tables: senna herbal tea, rhubarb root herbal tea, and green tea. The study was double-blinded, so researchers and intervention participants were unaware of the tea allocation until intervention and analysis completion.

### 2.2. Sample Size Calculation

Based on the information obtained from the research for LDL cholesterol (power of 80%, and a confidence interval of 95%) [40], the sample size was estimated to be 15–20 participants per group, accounting for an estimated 10% drop-outs. The effect size was calculated post trial. 

### 2.3. Study Design: Dose, and Type of Herbal Teas

Infusions were taken after the last meal/snack of the day (post 18:00 h) daily, for 21 days and no food was consumed afterward.

Senna herbal bags (total weight 2.5 g) contained the following ingredients: green tea 0.75 g from Dartmoor Estate Tea, UK [41], senna leaves (*Cassia angustifolia Vahl*) 1.2 g, alongside cassia seeds, lotus leaf, fiveleaf gynostemma, honeysuckle flower and hawthorn fruit, all at <0.33 g each. Rhubarb root herbal tea bags contained the same as the senna tea bags; however, 1.2 g dried Stockbridge arrow root (*Rheum rhabarbarum*) replaced the senna. Active control green tea bags contained 0.75 g of green tea only. Tea/herbal tea bags were infused in 190 mL of hot water (80–100 °C) by the participants at home and stirred clockwise 10 consecutive times to allow for optimal infusion and then allowed to brew for 5 min, before removal of the teabag and consumption of the infusion (infusions were standardised to 16.8 ± 2.5 ug/mL of green tea catechins, Table 1 and Supplementary Table S1.

### 2.4. Anthropometric Measures

Body weight (using SECA 799 Electronic Column Scales to the nearest 0.1 kg) and height was measured (using a Holtain Stadiometer to the nearest cm) to allow the BMI to be calculated and compared to ranges set out by the WHO [42]. Waist circumference (midway between the lower rib and the iliac crest on the midaxillary line) and hip (level of the widest circumference over the great trochanters) were taken to the nearest 0.1 cm, using an ergonomic circumference measuring tape over bare skin, whenever possible. Triplicate measurements were made, and the mean was calculated, allowing the waist-to-hip ratio to be calculated and compared to published ranges [43,44]. All measurements were taken in the morning by the researcher after the participants had been fasting for at least 8 h.

### 2.5. Food Recall and Stool Consistency

Diet was assessed using Prime Diet Quality Score (PDQS) [45](amended to account for vegetarian and vegan choices, providing an assessment of an individual’s diet and eating habits. Bristol stool scale [46] provided data concerning the consistency of the stool. Both questionaries were completed on the first and last day of the trial and were not researcher-led. All participants were requested to maintain their habitual physical activity. To evaluate compliance, participants were requested to note any missed teabags and to bring back the remaining teabags.

### 2.6. Collection, Preparation of Blood Samples and Analysis

Fasting bloods were collected by the participants fingers being pricked by the researchers using lancets and collected into BD Microtainer™ Tubes (600 μL) with Microgard™ Closure containing anticoagulant (Lithium heparin). Plasma was extracted by inversion and then centrifugation at 4000× *g* for 20 min at 4 °C. Plasma samples were analysed using the Afinion AS100 (Abbott, Abbott Park, IL, USA) lipid panel (total cholesterol, LDL cholesterol, HDL cholesterol, triglycerides, and non-HDL cholesterol).

Since participants had their lipid profiles measured before and after the intervention, these data were treated as paired measurements, and the dependency on participant identity was taken into account for statistical analysis. Age and BMI were included as covariates in the analysis to adjust for any potential effects related to these variables. Gender was not included as a covariate due to the highly unbalanced distribution of participants. As there was no prior information on the direction of the effect of the infusions (positive, negative, or neutral), we conducted tests for differences in lipid concentrations before and after the intervention.

To minimise Type I error inflation from multiple comparisons, we performed a repeated measures ANOVA using the base R stats package version 1.10.5 [47]. The model included participant ID as a random effect, and age and BMI as continuous covariates. Fixed effects included time (before and after the intervention), treatment group (green tea, rhubarb root, and senna infusion), and their interaction.

We analysed the six blood lipid measurements separately: total cholesterol, LDL cholesterol, HDL cholesterol, triglycerides, non-HDL cholesterol and HDL/LDL ratio.

Post hoc analyses were conducted using the R package emmeans [48] to calculate marginal means and estimate the differences in lipid concentrations resulting from the consumption of any experimental tea, regardless of type. We also tested for interaction effects to evaluate differences in lipid concentrations before and after the intervention for each infusion group.

### 2.7. Collection, Preparation of Stool Samples and Analysis

Faecal samples were collected and preserved in OMNIgene.GUT OM-200 (DNAgenotek, Ottawa, Canada) kits. DNA extraction was performed using the QIAamp PowerFecal Pro DNA Kit. For library preparation, the V4 hypervariable region of the 16S rRNA gene was amplified using specific primers as previously described [49]. Libraries were quality-checked and normalised using the Qubit HS DNA quantification kit before sequencing on an Illumina MiSeq platform at the Swansea University Medical School Sequencing Facility. Sequence processing was conducted with Mothur (version 1.44.1), clustering reads into operational taxonomic units (OTUs) at a 3% dissimilarity threshold to analyse community structure. Taxonomic classification utilised the SILVA release 132 reference database, and sequences identified as chloroplast, eukaryotic, mitochondrial, or archaeal were filtered out to refine the dataset. Alpha diversity metrics—including observed OTUs (Sobs), Shannon evenness, and the inverse Simpson diversity index—were calculated in Mothur. Statistical analyses and graphical representations were completed in RStudio using the tidyverse (version 1.3.2), phyloseq (version 1.40.0), and vegan (version 2.6.2) packages. Alpha diversity comparisons were conducted with pairwise Wilcoxon rank sum tests. To control for the false discovery rate (FDR) from multiple pairwise comparisons, *p*-values were adjusted using the Benjamani–Hochberg method. This approach ranks *p*-values and applies a correction factor based on the number of tests, reducing the likelihood of type I errors while maintaining statistical power. Adjusted *p*-values < 0.05 were considered statistically significant. To investigate correlations between bacterial genera and lipid concentrations (Cholesterol, LDL-cholesterol, HDL-cholesterol, triglycerides, non-HDL cholesterol), genus-level linear regression analyses were conducted. Genera relative abundances were calculated, and their associations with lipid measurements were assessed using Spearman’s rank correlation. *p*-values from these correlations were also adjusted using the Benjamani–Hochberg method to account for the large number of genera tested. Genera with significant correlations (adjusted *p* < 0.05) were further explored through linear regression to characterise these associations. Community-level (beta diversity) analyses used the Bray–Curtis distance metric. Differences in microbial composition between groups were assessed using adonis2 permutational multivariate analysis of variance with 100,000 permutations, accounting for confounding factors, including age and BMI, and interactions between sampling points (pre- and post-intervention). This enabled a robust assessment of community composition differences across intervention groups and time points. Data are available in NCBI BioProject as fastq files (PRJNA1182543).

## 3. Results

**Figure 1:**
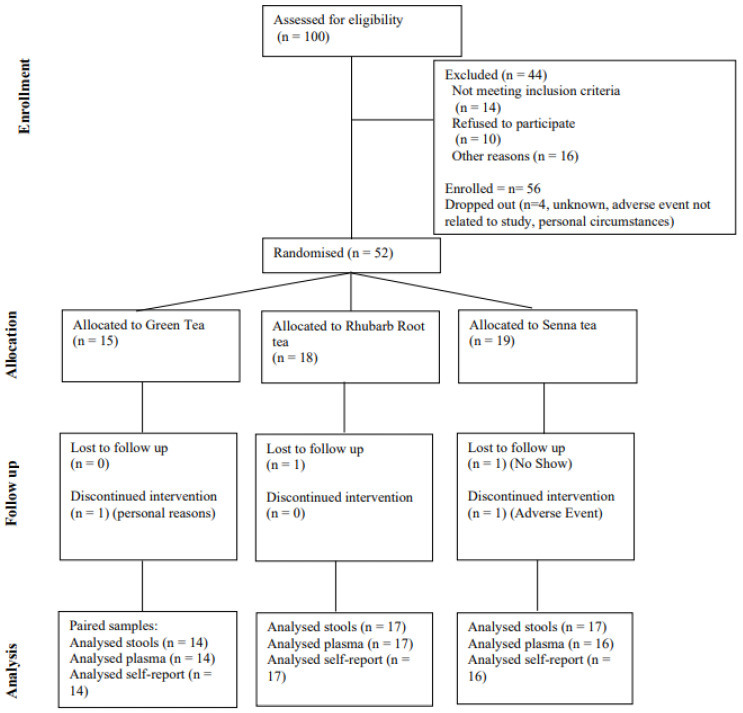
CNSORT diagram showing the flow of participants through each stage of the randomised trial.

A total of 56 participants who met the inclusion criteria were enrolled in the study. Four of the participants dropped out; therefore, 52 participants were randomised into green, rhubarb root herbal and senna herbal tea for a 21-day period (Figure 1 and Table 2). The mean age and BMI of the participants were 52.86 ± 12.53 (years) and 29.90 ± 5.83 (kg/m^2^), respectively. Based on the residual green tea in the packed sachets, participants had high adherence and compliance rate was over 98%, and no serious adverse events were associated with drinking the herbal tea. One participant reported an adverse event five days after the first intervention day; however, there was no evidence of any reasonable causal relationship to the trial intervention. The participant feedback was recorded, and they chose to withdraw from the study, reporting full health two days later. Eight individuals reported that they had an increase in bowel movements; however, none of these expressed this to be problematic, or a reason to stop participating.

The comparison between the diet score before and after the intervention showed no significant change between or within any of the trial arms. A similar outcome was shown with the BMI and waist/hips ratio, no changes were observed.

Because lipid profiles were measured before and after the intervention trial, the data are treated as repeated measurements, requiring consideration of sample dependence based on participant identity in the statistical analysis. Therefore, only the 47 participants who provided blood samples both before and after the trial were included in the analysis.

The analysis revealed that time had a highly significant effect on cholesterol concentrations (*p* = 0.0016), indicating a meaningful change in cholesterol over the course of the study. However, the interaction between treatment and time was not significant (*p* = 0.5891), suggesting that the effect of the treatments did not significantly differ over time. For individual group baseline to post-intervention comparisons, green tea showed a marginally significant change (*p* = 0.0587), suggesting a potential trend, but the evidence was not strong enough to draw firm conclusions. Rhubarb root herbal tea demonstrated a significant change (*p* = 0.0102), indicating a meaningful reduction in cholesterol, while senna herbal tea showed no significant change (*p* = 0.2474) from baseline to post-intervention.

Similarly, time had a highly significant effect on LDL-cholesterol concentrations (*p* = 0.0000), suggesting a meaningful change over time. However, the treatment x time interaction was not significant (*p* = 0.2910), indicating that the treatments did not have differing effects over time. For individual group baseline to post-intervention comparisons, green tea had a significant reduction in LDL-cholesterol (*p* = 0.0011), and rhubarb root herbal tea showed a highly significant reduction (*p* = 0.0006). In contrast, senna herbal tea showed no significant change in LDL-cholesterol (*p* = 0.0986). Age had a marginally significant effect on LDL-cholesterol (*p* = 0.0564), but this result was borderline significant, while BMI had no significant effect on LDL-cholesterol (*p* = 0.1903).

For non-HDL cholesterol, the results followed a similar pattern as LDL-cholesterol. Green tea and rhubarb root herbal tea both showed significant reductions in non-HDL concentrations (*p* = 0.0219 and *p* = 0.0112, respectively). In contrast, senna herbal tea showed no significant change in non-HDL cholesterol (*p* = 0.2042).

Finally, the analysis revealed that none of the treatments had a significant effect on triglyceride concentrations or HDL-cholesterol. For triglycerides, the *p*-values for green tea (*p* = 0.1920), rhubarb root herbal tea (*p* = 0.2503), and senna herbal tea (*p* = 0.7126) were all non-significant. Similarly, none of the treatments significantly affected HDL-cholesterol (green tea *p* = 0.7903, rhubarb root tea *p* = 0.6207, and senna tea *p* = 0.8958).

The rhubarb herbal tea group showed a large effect in LDL cholesterol, non-HDL cholesterol, and the Chol/HDL ratio, all greater than −0.8. Additionally, the rhubarb herbal tea group had sufficient statistical power (0.91517), while the green tea groups fell below the threshold for adequate power.

Table 3 and full data in Supplementary Table S3 present the mean differences in chemical concentrations between baseline and post-intervention for each group-chemical combination, along with the standard deviation of these differences.

We evaluated the effects of tea formulation on faecal alpha diversity metrics—Shannon evenness, observed species richness, and the inverse Simpson index—across pre- and post-intervention samples in 48 paired samples, accounting for age as a covariate. Age was significantly associated with higher microbial evenness, richness, and diversity. Shannon evenness (*p* = 0.010), species richness (*p* = 0.005), and inverse Simpson index (*p* = 0.013) all increased with age. While the models explained a modest proportion of variation (R^2^ = 0.054–0.073), these results suggest that microbial communities become more stable and diverse as individuals age. While minor, non-significant trends were observed for tea formulations, the tea formulations tested did not significantly alter alpha diversity over the intervention period.

**Figure 2:**
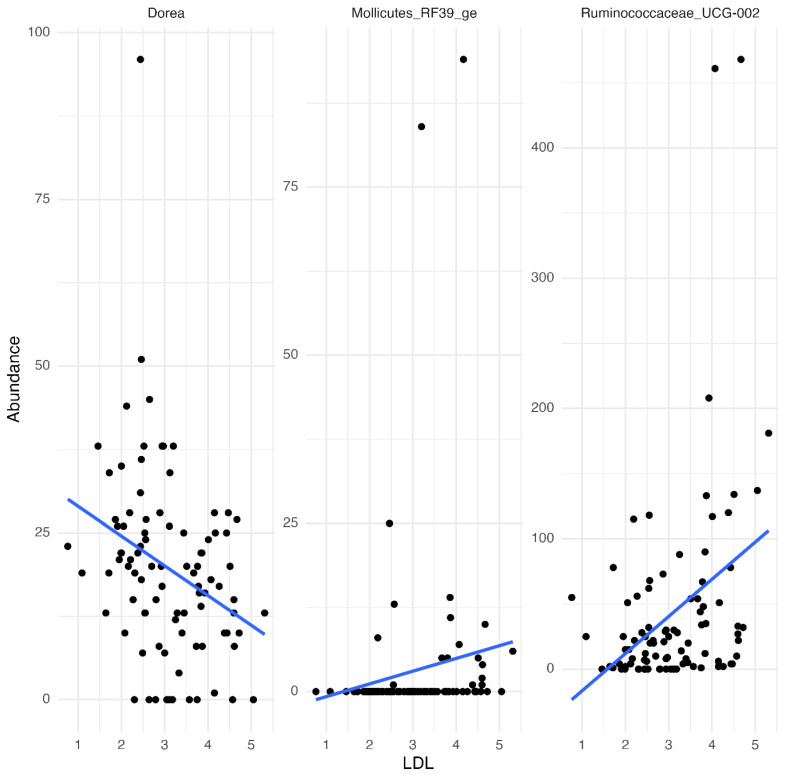
Linear regression models illustrate significant correlations (*p*.adjust < 0.05) between LDL concentrations and the relative abundances of bacterial genera, including Dorea, Mollicutes RF39_ge, and Ruminococcaceae UCG-002.

We assessed correlations between specific genera abundances and lipid profile components, focusing on LDL-cholesterol concentrations. Using linear modelling, we identified significant correlations (*p*.adjust < 0.05) between LDL-cholesterol concentrations and the abundance of several bacterial genera. Notably, *Dorea* spp. abundance was inversely associated with LDL-cholesterol, suggesting a potential protective effect or inverse relationship, as higher *Dorea* abundance correlated with lower LDL-cholesterol concentrations. In contrast, positive correlations were observed between LDL-cholesterol concentrations and both *Mollicutes RF39ge* and *Ruminococcaceae UCG-002*, where higher abundances in these genera were associated with elevated LDL-cholesterol (Figure 2).

The blue lines represent fitted regression models, highlighting the direction and strength of the relationships. Statistical significance was determined using Spearman’s rank correlation with Benjamani–Hochberg *p*-value adjustments.

Despite these genus-level correlations, community-level analyses using adonis2, accounting for potential confounding factors such as age or BMI and interactions between sample points, revealed no significant differences in overall microbial composition between pre- and post-intervention samples within any of the three tea groups, even with 100,000 permutations. This suggests that, while specific bacterial genera correlate with LDL-cholesterol changes, the broader microbial community structure remains stable across the intervention period, unaffected by these other confounding factors. Notably, no detrimental shifts in microbial composition were detected over the intervention period, suggesting that gut balance was preserved without any adverse impacts in this healthy human cohort.

## 4. Discussion

The findings from this study provide evidence supporting the lipid-lowering effects of green tea with rhubarb root and other herbs while preserving gut microbiota balance. The significant reduction in LDL cholesterol, total cholesterol, and non-HDL cholesterol observed in the rhubarb root group aligns with previous studies on the hypolipidemic effects of anthraquinones, including those present in rhubarb [32,50]. Notably, these reductions were achieved without adverse impacts on gut microbial diversity, indicating potential for these teas to support cardiovascular health without disturbing gut homeostasis.

Comparisons with existing research reveal both consistencies and novel contributions. The observed LDL cholesterol reductions in the green tea group support prior meta-analyses showing green tea’s effectiveness in improving lipid profiles [22,23]. However, the rhubarb root tea demonstrated even greater lipid-lowering efficacy, suggesting an additive or synergistic effect of rhubarb anthraquinones with green tea catechins. Interestingly, senna tea, despite its known pharmacological properties, did not significantly impact lipid concentrations. This finding contrasts with rodent studies that reported improvements in lipid profiles after senna injection [26]. The difference may be due to the unique physiological responses in humans or the relatively short intervention duration in this study.

This study highlights the ability of green tea with rhubarb root to preserve gut microbial stability while causing reductions in plasma LDL cholesterol. Correlations between *Dorea* spp. abundance and LDL cholesterol concentrations provide novel insights into potential microbiota-mediated mechanisms, suggesting that specific bacterial genera may influence lipid metabolism [24]. These findings align with prior research showing that short-term green tea consumption has a minimal impact on overall microbial diversity while supporting beneficial microbial interactions [24]. The inverse relationship between *Dorea* spp. and LDL cholesterol indicates a potential protective role in lipid regulation, whereas positive associations with *Mollicutes RF39ge* and *Ruminococcaceae UCG-002* suggest possible links to dyslipidaemia that warrant further exploration. Maintaining microbial eubiosis alongside lipid-lowering effects underscores the functional potential of herbal teas [50].

While this study primarily highlighted the significant reductions in LDL cholesterol, total cholesterol, and non-HDL cholesterol achieved with rhubarb root tea, triglycerides were also evaluated as part of the lipid profile. Elevated triglycerides are a well-established risk factor for cardiovascular diseases, contributing to a pro-inflammatory and pro-thrombotic state that exacerbates vascular risk [49,51]. However, in this study, none of the tea interventions, showed significant effects on triglyceride concentrations. These findings align with some previous short-term dietary interventions, where triglyceride change often requires longer or intensive interventions [52,53].

While this study provides promising evidence supporting the lipid-lowering effects of green tea with rhubarb root and its ability to maintain gut microbial stability, there are limitations. The lack of significant change in triglycerides may be attributed to the relatively short 21-day duration of the trial, which may not have been sufficient to cause detectable improvements. Additionally, participants’ baseline lipid concentrations were within normal ranges, potentially limiting the magnitude of observable changes. Furthermore, the study was conducted in a free-living, healthy human cohort, which, although reflective of real-world conditions, may have introduced variability in dietary adherence and lifestyle factors. The small sample size, particularly within each treatment group, may have reduced the power to detect changes in parameters such as HDL cholesterol and triglycerides or to generalise findings to broader populations. Additionally, the tea formulations contained additional herbal ingredients, including cassia seeds, lotus leaf, fiveleaf gynostemma, honeysuckle flower, and hawthorn fruit, which may have contributed to the observed effects. Although green tea and rhubarb root were the primary components studied, the potential additive or synergistic effects of these other botanicals cannot be ruled out.

Future studies could investigate these teas in populations with hypertriglyceridemia or extend the intervention duration to better assess their potential benefits on lipid concentrations. Larger, multi-centre trials are also warranted to confirm these effects and evaluate their applicability across diverse demographic and clinical groups. Further research isolating the impact of individual ingredients is necessary to fully outline their specific contributions. Finally, while the study observed correlations between specific bacterial genera and lipid profiles, causality cannot be inferred from these associations. Future mechanistic studies employing metabolomics, metagenomics, and controlled dietary interventions are necessary to clarify the pathways involved in microbiota-mediated lipid metabolism.

## 5. Conclusions

This study highlights the potential of rhubarb root herbal tea as a functional beverage for improving lipid profiles while maintaining gut microbial balance. The findings suggest that daily consumption of rhubarb root herbal or green tea over 21 days can significantly lower LDL cholesterol and total cholesterol without adverse effects on gut health. These results provide a foundation for future research to explore the long-term benefits and mechanistic underpinnings of these herbal teas, potentially informing dietary strategies for cardiovascular disease prevention.

By integrating traditional herbal ingredients with modern clinical research, this study underscores the importance of investigating functional foods to address prevalent health concerns such as CVD and dyslipidaemia. Future studies should aim to replicate these findings in larger, more diverse cohorts and explore the interaction of these teas with other dietary and lifestyle interventions.

## Figures and Tables

**Table 1:** Mean content of epistructured catechins in the green tea infusion.

Catechins	Absolute	Relative (% Total Catechins)
	(ug/mL Tea Infusion)	
Epigallocatechin (EGC)	8.4 ± 1.9	50.0
Epicatechin (EC)	5.2 ± 0.5	31.2
Epigallocatechin gallate (EGCG)	2.2 ± 0.2	12.8
Epicatechin gallate (ECG)	1.0 ± 0.2	6.0
Total	16.8 ± 2.5	100

**Table 2:** Characteristics of the study participants at baseline.

Variable	Mean	(SD)
Demographics		
Total taking part in study [n]	52	
Gender Female [n]	45	
Gender Male [n]	7	
Age [years]	52.86	12.53
Age range (min–max) [years]	21–71	
Anthropometrics		
Weight [kg]	82.25	17.66
Height [cm]	165.68	8.48
BMI [kg m^−2^]	29.90	5.83
Waist circumference [cm]	97.13	15.12
Hip circumference [cm]	112.94	11.94
Waist to Hip ratio	0.86	0.08
M	0.93	0.05
F	0.85	0.16

Where BMI: body mass index.

**Table 3:** ANOVA of lipid concentrations between post- and pre-intervention for each group-chemical combination, along with the standard error of these differences.

Lipid Group	Interaction	Df	Estimate	SE	t.ratio	*p*.Value
Cholesterol	Green tea: Baseline—post intervention	44	−0.2280	0.1170	−1.9410	0.0587
	Rhubarb root herbal tea: Baseline—post intervention	44	−0.2860	0.1070	−2.6830	0.0102 *
	Senna herbal tea: Baseline—post intervention	44	−0.1290	0.1100	−1.1720	0.2474
Triglycerides	Green tea: Baseline—post intervention	44	−0.2280	0.1170	−1.9410	0.1920
	Rhubarb root herbal tea: Baseline—post intervention	44	−0.2860	0.1070	−2.6830	0.2503
	Senna herbal tea: Baseline—post intervention	44	−0.1290	0.1100	−1.1720	0.7126
HDL-cholesterol	Green tea: Baseline—post intervention	44	−0.2280	0.1170	−1.9410	0.7903
	Rhubarb root herbal tea: Baseline—post intervention	44	−0.2860	0.1070	−2.6830	0.6207
	Senna herbal tea: Baseline—post intervention	44	−0.1290	0.1100	−1.1720	0.8958
LDL-cholesterol	Green tea: Baseline—post intervention	44	−0.2280	0.1170	−1.9410	0.0011 **
	Rhubarb root herbal tea: Baseline—post intervention	44	−0.2860	0.1070	−2.6830	0.0006 **
	Senna herbal tea: Baseline—post intervention	44	−0.1290	0.1100	−1.1720	0.0986
Cholesterol-HDL ratio	Green tea: Baseline—post intervention	44	−0.2280	0.1170	−1.9410	0.0843
	Rhubarb root herbal tea: Baseline—post intervention	44	−0.2860	0.1070	−2.6830	0.4957
	Senna herbal tea: Baseline—post intervention	44	−0.1290	0.1100	−1.1720	0.1421
non HDL-cholesterol	Green tea: Baseline—post intervention	44	−0.2280	0.1170	−1.9410	0.0219 **
	Rhubarb root herbal tea: Baseline—post intervention	44	−0.2860	0.1070	−2.6830	0.0112 **
	Senna herbal tea: Baseline—post intervention	44	−0.1290	0.1100	−1.1720	0.2042

where Df, Degrees of Freedom; Estimate, the estimated mean difference between groups being compared. SE, Standard Error): t.ratio, t-statistic; *p*.value < 0.05 **, *p* < 0.01 * indicates significant differences.

## Data Availability

Datasets are available on request and in Supplementary Table S2. Raw FASTQ files generated in this study have been deposited in the NCBI SRA and are accessible through the BioProject database under accession number PRJNA1182543. Any other raw data supporting the conclusions of this article can be made available by the authors, without undue reservation.

## Supplementary Materials

The following supporting information can be downloaded at: https://www.mdpi.com/article/10.3390/metabo15020139/s1, Supplementary Table S1: Optimised LC-MS/MS parameters of epistructured catechins in the green tea infusion. Supplementary Table S2: Full lipid concentration data. Supplementary Table S3: Full ANOVA tables presenting the mean differences in chemical concentrations between post- and pre-intervention for each group-chemical combination. Where Df, Degrees of Freedom; Sum Sq, Sum of Squares; Mean Sq, Mean Square; F value, ratio of two Mean Squares, Pr(>F), *p*-value; Estimate, the estimated mean difference between groups being compared; SE, Standard Error; t.ratio, t-statistic; *p*.value ≤ 0.001 ***, *p* < 0.05 **, *p* < 0.01 * indicates significant differences. Refs. [54,55,56] are cited.

## Abbreviations

CVD, cardiovascular disease; LDL, low-density lipoprotein; HDL, high-density lipoprotein; non-HDL, non-high-density lipoprotein.
